# Cone beam computed tomography (CBCT) guidance is helpful in reducing dose exposure to pediatric patients undergoing radiofrequency ablation of osteoid osteoma

**DOI:** 10.1007/s11547-021-01439-4

**Published:** 2021-12-27

**Authors:** Francesco Fiore, Francesco Somma, Roberto D’Angelo, Luca Tarotto, Vincenzo Stoia

**Affiliations:** 1 Radiologia Interventistica, Istituto Nazionale Tumori IRCCS “Fondazione G. Pascale”, Via Mariano Semmola 52, 80131 Napoli, Italy; 2 Medicina Nucleare, Policlinico Universitario Di Bari, Piazzale Giulio Cesare 11, Bari, Italy

**Keywords:** Osteoid osteoma, Thermal ablation, Radiofrequency ablation, CT, CBCT, Fluoroscopy

## Abstract

**Purpose:**

To assess efficacy and safety of cone beam computed tomography (CBCT) in the radiofrequency ablation (RFA) of osteoid osteoma (OO) in children and adolescents, and to compare technical success, clinical success, radiation dose and procedure duration time of CBCT guidance to conventional computed tomography (CT) guidance.

**Materials and methods:**

Between 2015 and 2019, 53 consecutive percutaneous RFA were performed on pediatric patients with CBCT or conventional CT guidance, respectively, in 24 and 29 children and adolescents with 24-month follow-up. Dose area product (DAP) and dose length product (DLP) were recorded, respectively, for CBCT and conventional CT and converted to effective doses (ED).

**Results:**

CBCT and conventional CT groups were similar in terms of patient age and weight, tumor size and tumor location. Technical success was achieved in all cases. Primary clinical success was 91.67% (22/24) for the CBCT group and 89.66% (26/29) for the conventional CT group. Mean DAP was 64.75Gycm2 (range 6.0–266.7). Mean DLP was 972.62mGycm (range 337–2344). ED was significantly lower in the CBCT group compared to the conventional CT group (0.34 mSv vs. 5.53 mSv, p = 0.0119). Procedure duration time was not significantly longer in the CBCT group (102.25 min vs. 92.34 min, *p* = 0.065). No major complication was registered. Minor complications were observed in 4 patients (2 in CBCT; 2 in conventional CT).

**Conclusions:**

Compared to conventional CT guidance, CBCT guidance for percutaneous OO ablation shows similar technical and clinical success rates, with reduced radiation dose and equivalent procedure duration time. This technique helps sparing dose exposure to pediatric patients.

## Introduction

Osteoid osteoma (OO) is a benign bone tumor that commonly arises in children and adolescents and is slightly predominant in male population [1]. Generally, it is seen as a little central nidus surrounded by peripheral reactive zone of osteoblasts and thickened cortical bone and fibrotic tissue with vascular elements [2]. The radiographic appearance is typical and consists of a small central radiolucent nidus with bony sclerosis all around, with cortical thickening caused by subperiosteal bone formation [3]. Nowadays, Computed Tomography (CT) is the gold-standard to reach the diagnosis, showing the precise location and size of the nidus. Imaging-guided percutaneous thermal ablation techniques, such as radiofrequency ablation (RFA), cryoablation, microwave ablation (MWA), laser photocoagulation and Magnetic Resonance-guided Focused Ultrasound (MRgFUS), became the first line treatment [4–8]. In particular, CT-guided RFA is the most common technique among percutaneous treatment of OO at present. Accurate placement of the ablation needle is crucial for a complete nidus ablation, even if hard-to-access locations or to low patient compliance make it very challenging and time consuming in certain patients. Limiting exposure of patients to ionizing radiation is necessary in all patients and mandatory in children and adolescents, thus forcing radiologists to lower radiation exposure in case of OO ablation.

Cone beam computed tomography (CBCT) is a new technique that has increasingly been used for imaging-guidance during interventional procedures, including percutaneous bone and muscle tumor ablation [9–13]. Indeed, software allows the CBCT volumetric data to be superimposed on live fluoroscopy, drawing a trajectory for the procedure to be realized under real-time fluoroscopy planning and monitoring [14].

Aim of this study is to assess efficacy and safety of CBCT in the thermal ablation of OO in children and adolescents, and to compare technical success, clinical success, radiation dose and procedure duration time of RFA with CBCT guidance to RFA with conventional CT guidance.

## Material and methods

### Patients population

Between March 2014 and March 2019, 55 consecutive pediatric patients were evaluated in our Interventional Radiology Department for chronic bone pain with nocturnal exacerbation relieved by salicylates and eventually diagnosed with OO based on radiographic, CT and/or magnetic resonance imaging (MRI) findings and clinical history. Two of them were not eligible to percutaneous thermal ablation because of platelet count < 50,000/µL (1) and coagulation disorder (1). The remaining 53 underwent RFA using CBCT with fluoroscopic guidance overlay (24) or using conventional CT guidance (29). The choice of CBCT versus conventional CT was due to the fact that the CBCT technology was available in our department from the end of 2017, so that the vast majority of CBCT procedures performed in 2018 and 2019, and the majority of conventional CT cases were performed between 2014 and 2017.

### Cone beam CT-guided procedure

No patient was treated under general anesthesia. Peripheral nerve anesthesia was conducted under ultrasound guidance in patients with OO in the extremities. Local anesthesia (2–5 ml of mepivacaine hydrochloride at 2%) was performed in the cutaneous site of needle penetration. In some cases, subdural anesthesia was performed. An unenhanced CBCT of the anatomic region was performed, and images were reviewed on an adjacent workstation in order to identify target site and skin entrance area on multiplanar reconstructions (MPR). Probe placement trajectory was determined using a navigation system software (SIRIO, Masmec manufactured). The patients are generally placed around one meter under the navigation system arm. A square plastic device with four little spheres in the corners is applied on the insertion body region, and a CT scan is performed so that the insertion point is acquired by the navigation system. Then, a similar plastic device with four spheres is applied on the needle and recognized by the navigation system, which processes and provides the right trajectory to the lesion and a real-time tracking of the advancing needle. After a stab incision, the biopsy trocar (Bone Biopsy System, Bonopty, Radi Medical Device, Sweden) was advanced under intermittent fluoroscopic guidance just into the bone cortex superficial to the lesion. Drilling through the nidus under live fluoroscopic guidance with overlay on CBCT images was mandatory in cases of dense cortical bone (Bonopty drill set, standard length 122 mm, extended length 160 mm, caliber 17 G12/ 1.7-mm). After reaching the nidus, tissue sample was obtained for histology using a biopsy needle (Bonopty biopsy needle, Radi Medical Device, Sweden) inserted through the trocar. Next, the electrode was inserted through the trocar aiming at the center of the nidus of its active tip under imaging-guidance. A RF bipolar ablation system (Covidien) was used to perform RFA by raising the temperature directly to 90–93 °C for around 6 min.

### Conventional CT-guided procedure

No patient was treated under general anesthesia. Conventional CT-guided procedures were performed on a GE Optima 64-slice CT (GE Medical Systems, Milwaukee, WI). After administration of local anesthesia (2–5 ml of mepivacaine hydrochloride at 2%) in the cutaneous site of needle penetration, unenhanced CT with multiplanar reconstruction (MPR) was used to localize the lesion and plan the best approach to avoid vital structures. Then, the position was ascertained by CT using a radiopaque landmark, and the skin was marked. A stab incision was made, and a biopsy trocar (Bone Biopsy System, Bonopty, Radi Medical Device, Sweden) was advanced to cortex. Drilling through the cortex was mandatory in cases of dense cortical bone (Bonopty drill set, standard length 122 mm, extended length 160 mm, caliber 17 G12/ 1.7-mm). Once the probe reached the target, a final CT scan was performed in all cases to document probe position, and a lesion tissue sample was obtained for histology using a biopsy needle (Bonopty biopsy needle, Radi Medical Device, Sweden) inserted through the trocar. Next, the electrode was inserted through the trocar aiming at the center of the nidus of its active tip under imaging-guidance. A RF bipolar ablation system (Covidien) was used to perform RFA by raising the temperature directly to 90–93 °C for around 6 min. The total number of scans and images performed varied on the base of the operator’s needs. The total number of scans to reach the lesion varied on the base of the lesion location.

### Definitions

Technical success was defined as the correct placement of the electrode in the lesion, so that no portion of the lesion was more than 5–7 mm away from the exposed tip [15]. Clinical success was considered as the complete disappearance of symptoms after the treatment. Residual symptoms were defined as pain or impaired function or both identical to the presenting complaints that persisted for more than 2 weeks after radiofrequency thermal ablation. Recurrent symptoms were defined as the reappearance of symptoms that followed a symptom-free period after RF thermal ablation [16].

### Outcome measures

Technical success was considered as the primary outcome in this study. Clinical success, reintervention rate, complications, radiation dose and procedure duration time were also recorded as secondary outcomes. Visual Analog Scale (VAS) for numeric pain score (0–10) was recorded at presentation and during follow-up. Response to analgesics was also recorded.

Primary clinical success was defined as VAS of 1.5/10 or less after a single ablation. Secondary clinical success was defined as VAS of 1.5/10 or less following repeated ablations. Major complications were defined as injuries requiring further therapy, hospitalization, permanent adverse sequelae or death. Minor complications were defined as injuries requiring no further therapy, with no permanent adverse sequelae.

Radiation dose for each procedure was estimated by collecting dose area product (DAP) and dose length product (DLP) and converting them to effective dose (ED) for comparison. The following published conversion factors were considered: (a) for DAP to ED conversion, established conversion coefficients based on phantom models were used, ranging from 0.0034 to 0.0101 mSv/mGy * cm^2^ [17], considering patient age and area of body scanned [18, 19]; (b) for DLP to ED conversion, established conversion coefficients (k-factors) based on phantom models were used, ranging from 0.0003 to 0.0271 mSv/mGy * cm [17], considering patient age and area of body scanned. The procedure duration time was defined considering the patients’ arrival time in the interventional radiology room and the exit time from the same room.

### Statistical analysis

The statistical analysis was performed with MATLAB statistical toolbox version 2008 (MathWorks, Natick, MA, USA) for Windows at 32 bit. Data were categorized as numbers and percentage for qualitative variables and mean and range for quantitative variables. Groups characteristics were compared through Chi-squared test for qualitative variables (gender, tumor location) and with Student’s t test for quantitative variables (age, tumor size). All tests with *p* value (*p*) < 0.05 were considered significant.

## Results

Tables 1 and 2 show the demographics of the patient population and the tumor characteristics, respectively.

**Table 1:** Demographics of the patient population

Parameters	Overall (*n* = 53)	Cone Beam CT (*n* = 24)	Conventional CT (*n* = 29)	*p *value
*Age*				
(years), mean ± SD	16.35 ± 2.22	16.08 ± 2.38	16.59 ± 2.09	0.42
*Weight*				
(kg), mean ± SD	55.30 ± 13.13	52.63 ± 11.56	57.52 ± 14.12	0.18
*Gender* (%)				
Male	36/53 (67.92)			
Female	17/53 (32.08)			
*Imaging for Diagnosis* (%)				
CT	37/53 (69.81)			
MRI	11/53 (20.75)			
MRI + CT	5/53 (9.43)			
Tc 99-HDP Bone Scintigraphy *	3/53 (5.66)			
*Medicaments* (%)				
Aspirin	35/53 (66.04)			
Ibuprofen	17/53 (32.08)			
Naproxen	8/53 (15.09)			
Others	5/102 (4.90)			
*Pain duration before treatment*				
(months), mean ± SD	4.21 ± 1.84			

*SD* standard deviation, *CT* Computed Tomography, *MRI* Magnetic Resonance Imaging

^*^, in association with CT and/or MRI

**Table 2:** Tumor characteristics sorted by guidance technique (Cone Beam CT, conventional CT)

	Cone Beam CT (*n* = 24, %)	Conventional CT (*n* = 29, %)	Total (*n* = 53, %)	*p *value
*Location of the Lesion*				0.13
Femur	10 (18.87)	13 (24.53)	23 (40.35)	
Tibia	8 (15.09)	9 (19.98)	17 (32.08)	
Radius	1 (1.89)	2 (3.77)	3 (5.66)	
Iliac spine	2 (3.77)	0	2 (3.77)	
Acetabulum	1 (1.89)	1 (1.89)	2 (3.77)	
Sacrum	0	1 (1.89)	1 (1.89)	
Iliac crest	1 (1.89)	0	1 (1.89)	
Pedicle of L1	0	1 (1.89)	1 (1.89)	
Transverse process of L5	0	1 (1.89)	1 (1.89)	
Fibula	1 (1.89)	0	1 (1.89)	
Ulna	0	1 (1.89)	1 (1.89)	
*Side of the Lesion*				0.36
Left	13 (24.53)	17 (32.08)	30 (56.60)	
Right	10 (18.87)	13 (24.53)	23 (43.40)	
*Size of the Lesion*				0.22
Mean(mm) ± SD	9.96 ± 1.34	10.24 ± 1.66	10.11 ± 1.59	

*L* lumbar vertebra, *SD* standard deviation

A total of 53 lesions underwent RFA in 53 patients. Twenty-four tumors were ablated using fluoroscopic CBCT guidance, and 29 were ablated using conventional CT guidance. These groups were similar in terms of patient age and weight, tumor size (*p *value 0.22), tumor location (*p* value 0.13) and side of the lesion (*p* value 0.36). The average age was 16.08 years (range: 9–19 years) for CBCT group and 16.59 years (range: 11–19 years) for conventional CT group. The average weight was 52.63 kg (range: 35–78 kg) for CBCT group and 57.31 kg (range: 39–84 years) for conventional CT group. The average size of the lesions was 9.96 mm (range: 6–21 mm) for CBCT group and 10.24 mm (range: 7–19 mm) for conventional CT group. The mean duration of symptoms before ablation was overall 4.21 months (range: 1–8 months, standard deviation: 1.84).

Outcomes are showed in Table 3.

**Table 3:** Outcomes sorted by guidance technique (Cone Beam CT, conventional CT)

	Cone Beam CT (*n* = 24, %)	Conventional CT (*n* = 29, %)	Total (*n* = 53, %)	*p* value
Technical success	24/24 (100%)	29/29 (100%)	53/53 (100%)	0.13
Clinical success	24/24 (100%)	29/29 (100%)	53/53 (100%)	0.36
Major complications	0	0	0	
Minor complications	2/24 (8.33%)	2/29 (6.89%)	4/53 (7.55%)	
Procedure duration time (min, range)	102.25 (72–145)	92.34 (64–137)		0.65
Effective dose (mSv)	0.34 (0.02–1.51)	5.53 (0.27–34.54)		0.0119

min, minutes

Technical success of 100% was registered in both groups. Primary clinical success was 91.67% (22/24) for the CBCT group and 89.66% (26/29) for the conventional CT group. Secondary clinical success was 100%. Five cases of clinical failure were observed at one-month follow-up (three in the conventional CT group, two in the CBCT group). All these patients were retreated with RFA using the same guidance of the first procedure. None of them needed surgery. In case of CBCT guidance, the mean fluoroscopy time per procedure was 8.1 min (range 3.1–18.7) with a mean DAP of 64.75 Gy * cm2 (range 6.0–266.7). In case of conventional CT guidance procedures, a mean DLP of 972.62 mGy-cm (range 337–2344) was registered. Estimated effective radiation doses were significantly lower in the CBCT group compared to the conventional CT group (0.34 ± 0.40 vs. 5.53 ± 9.74 mSv, *p* = 0.0119).

The procedure duration time was not significantly longer in the CBCT group compared with the conventional CT group (102.25 vs. 92.34 min, *p* = 0.065). Post-ablation average duration of pain medication use was 1.1 days (range: 0–7 days, SD: 1.8 days) for CBCT group and 1.1 days (range: 0–7 days, SD: 1.8 days) for conventional CT, and by 1 week post-procedure, all patients had ceased medical therapy.

The procedure was overall well-tolerated. There was no major complication in the CBCT group nor in the conventional CT one. Thermal skin injury with subsequent resolution was described as a minor complication in one patient treated under CBCT guidance, and one treated under conventional CT guidance. Transient foot numbness was complained by one patient in the conventional CT group. Eventually, slight bone infection was found in one patient in the CBCT group and underwent pharmacological treatment. Overall mean follow-up time was at least 24 months (range: 24–31 months).

## Discussion

Some previous reports have described successful non-operative treatment of OO [20–22]. Nevertheless, the persistence of symptoms forces many patients to undergo definitive surgical or interventional ablative treatment. At present, percutaneous thermal ablation is considered as the standard of treatment in children and adolescents with OO, and RFA is the largely preferred ablative technique in children [23, 24]. Traditionally, CT guidance has been used to perform OO ablation procedures, sometimes with fluoroscopy. On the other hand, C-arm CBCT is a new imaging method that allows acquisition of cross-sectional imaging through the use of using modern flat panel detector angiographic systems. Probe placement trajectory can be planned and monitored thanks to volumetric images superimposed and co-displayed with fluoroscopic imaging in real-time [25]. Recently, the spreading of this newer technology has increased the use of CBCT in many interventional procedures [9, 12, 26, 27].

Compared to conventional CT, the use of CBCT allows a decreased radiation exposure to patients and operators, as showed by results in our series. This fact was proved through the direct comparison of the radiation dose administered to patients during OO ablation. In order to compare different radiating modalities, the recorded radiation output (DLP for conventional CT and DAP for CBCT) was converted to ED, which is generally used to account for the biological effects of radiation. This parameter represents an average dose to tissues adjusted according to organ-weighting factors proposed by the International Commission on Radiological Protection (ICRP) Publication 60 [28].

In recent years, “Image Gently” and “Step Lightly” campaigns well underlined the great importance of limiting radiation exposure as low as possible in pediatric patients [29, 30]. These campaigns comply with the ICRP Publication 60, which indicates the ED as an effective tool for the direct comparison of radiation dose due to different modalities. The ED values in both groups of our series are quite low, probably due to the fact that the great majority of the tumors were in the limbs, which are less radiosensitive than the trunk. This is due to the presence of many soft organs in thorax, abdomen and pelvis, thus increasing the ED conversion factors for these regions. This was an important result due to the fact that limiting ionizing radiation exposure and application of as low as reasonable achievable (ALARA) principles are crucial, particularly in the young patient population in whom OO frequently occurs. Nevertheless, this parameter should be constantly highlighted in the medical report of the procedure in order to avoid medicolegal litigations based on radiation exposure [31]. So far, the only radiation-free ablation technique described for OO is MRgFUS, a needless method that uses the ablative power of ultrasounds, with similar clinical outcome than RFA according to a recent paper by Arrigoni [8].

In patients undergoing OO ablation using conventional CT guidance, our mean DLP of 972.62 mGy-cm (range 337–2344) is markedly greater than what reported by Perry et al. in 2017 (61.5 mGy-cm) [32], but lower in comparison with the values reported by Cheng et al. in 2014 (up to 1058.8 mGy-cm) [33]. This is certainly due to the different lesion location: the great majority of the tumors in the series by Perry et al. [32] were located in the lower extremities (femur, tibia, or foot), which are composed of less radiosensitive tissues, while our series included lesions in trunk and pelvis. On the other side, the difference in comparison to the series by Cheng et al. [33] is probably due to low-dose protocols adopted by our technicians in case of pediatric patients.

Also in patients undergoing OO ablation using CBCT guidance, our mean DAP of 64.75 Gy * cm2 is greater than the one reported in previous published series including only OO in the limbs [32].

Well established conversion coefficients [17–19] based on phantom models were used to transform DAP and DLP in ED, thus allowing a comparison between different modalities.

Our mean conventional CT ED of 5.53 mSv (0.27–34.54) is definitely higher than previous published EDs for OO in the limbs (foot, 0.07 ± 0.05; knee, 16 ± 0.12; hip, 3.09 ± 1.37) [34], and slightly smaller than previous series considering whole body lesion locations.

Our mean CBCT ED of 0.34 mSv (0.019–1.51) is slightly superior to previous published values of 0.01–0.15 mSv [35, 36] for patients with OO located only in the extremities.

In general, our study supports the available literature that CBCT offers radiation dose reduction compared to conventional CT in the interventional ablation treatment of OO, which is of great importance in case of young patients.

With regard to the procedure duration time, a systematic review of the literature on RFA found an average ablation time of 6.8 min for treatment of OO [37]. This is in accordance with the ablation time registered in our series, both for CBCT and CT guidance groups. Differently, the total procedure duration time showed a slightly predominance of CBCT procedure, which was not statistically significant (102.25 vs. 92.34 min, *p* = 0.065). This parameter was intended as the total time spent between patient arrival in the interventional room and patient transfer to his hospital room after the procedure, thus including pre- and post-procedure preparation time. Although not statistically significant, this difference was likely due to a wrong practice in the use of CBCT software during the first three cases of OO, which caused a little prolongation of the procedure without any consequence for the patient. The correction of the wrong practice made the following CBCT procedures as fast as the conventional CT one. Overall, this result is different from what reported by Perry et al. [32], who found the total room utilization time for CBCT significantly greater than with conventional CT guidance and explained this fact with the learning curve for the use of the XperGuide software. A possible reason of this difference is that all procedures in our series were performed in local anesthesia while all procedures in the series by Perry et al. were performed in general anesthesia, thus reducing the patient preparation time in our study compared to the Perry’s one. Another reason is the fact that all interventions in our series were based on clinical and imaging diagnosis, and all diagnoses were histologically confirmed due to intraprocedural biopsy. This was performed in every patient before RFA, in order to disclose any other bone diseases mimicking OO, as described by some authors [38, 39].

With regard to the follow-up time, previous studies showed that recurrences of OO following thermal ablation mostly occur within the first year after the treatment. However, a recent series documented recurrence of OO up to 1.4 years after initially successful RFA [20, 40]. Therefore, our long-term follow-up time of 24 months is largely adequate to assess the initial treatment response and to catch late recurrences, as well.

Despite some potential limitations (lower contrast resolution, more susceptibility to beam-hardening artifacts), CBCT guidance has the undoubtable advantage of sparing dose exposure to patients, mainly thanks to the guidance software providing a real-time trajectory with fluoroscopic overlay.

The clinical success rate of 100% in this series is comparable to similarly powered pediatric RFA [1]. There were no major complications in this study. Overall, the minor complication rate was 7.55%, slightly under the range of 9–22% detected by other studies describing ablative techniques for treatment of OO [32].

The primary limitations of this study are the retrospective design and the single center nature. An update of this study with more cases has already been projected because a larger sample size would add additional power to our results. Another limitation is the lack of indisputable conversion coefficients to compare the radiation dose for different guidance techniques (CBCT and conventional CT). Eventually, a study standardized for the choice of the guidance technique would be desirable in order to avoid any statistical bias.

Contrary, potential strengths of this paper are: (1) the inclusion of lesions both in extremities and in trunk / pelvis; (2) the study population larger than previous published papers on the same topic.

In conclusion, CBCT guidance in the RFA of OO in pediatric patients is safe, highly effective, technically sound and clinically successful as much as conventional CT, with decreased radiation dose administered to little patients and without significant augmentation of procedure duration time. All this makes CBCT with fluoroscopic overlay guidance advisable in the pediatric population undergoing thermal ablation of OO.
